# A Case of Disseminated Zoster in an Immunocompetent Patient

**DOI:** 10.7759/cureus.6286

**Published:** 2019-12-04

**Authors:** Emily Drone, Latha Ganti

**Affiliations:** 1 Emergency Medicine, University of Central Florida/Hospital Corporation of America/Graduate Medical Education Consortium and Osceola Regional Medical Center, Olrando, USA; 2 Emergency Medicine, Envision Physician Services, Orlando, USA

**Keywords:** herpes zoster

## Abstract

The incidence of herpes zoster, more commonly known as shingles, is approximately 1.2 million individuals per year, which has been increasing worldwide. While the most common presentation of zoster is a rash and acute neuritis (the pain associated with the rash) within one or more contiguous dermatomes, other more serious manifestations such as herpes zoster ophthalmicus, acute retinal necrosis, Ramsay Hunt syndrome (herpes zoster oticus), aseptic meningitis, pneumonitis, bacterial superinfection and disseminated zoster ought to be considered by the clinician. This case report serves as a reminder for the emergency physician to bear these very serious complications in mind during the evaluation of a patient with suspected herpes zoster infection.

## Introduction

Herpes zoster is caused by the reactivation of the latent varicella-zoster virus. The previously dormant virus travels from the dorsal root ganglion [1] and down the sensory nerves to the skin to cause the classic single, unilateral dermatome rash that is typically associated with a shingles infection. A viral prodrome is common, including headache, malaise, and photophobia followed by pain, paresthesias, and itching along a dermatomal distribution. The rash usually presents in one or two contiguous dermatomes on the thorax or face, and it does not cross the midline of the body. The rash appears as clusters of vesicles and papules on an erythematous base in roughly the same stages of development [2]. One of the feared complications of this disease is the aforementioned, disseminated herpes. A patient might have a few scattered lesions outside of their sharply demarcated and localized rash in about 33% of immunocompetent patients. However, once greater than 20 such lesions are identified outside these discrete areas of cutaneous involvement, a diagnosis of disseminated herpes zoster can be made. This occurs in 2% of the general population and about 15%-30% of cases in immunocompromised patients [2]. Once disseminated disease is identified, a concern is raised for possible unknown immunocompromised state as well as other significant complications. Thus, sometimes a fairly simple clinical diagnosis can give way to a longer and more concerning differential as well as patient disposition dilemma.

## Case presentation

 A 67-year-old woman with a past medical history of controlled hypertension and diabetes mellitus presented to the emergency department (ED) with a painful rash for approximately one week. She complained of a red rash on the left side of her lower abdomen that wrapped around to her lower left flank. The rash was also present on her right anterior chest with some other similar lesions below her left eye and the right side of her chin. She further described the rash as blisters that are exquisitely painful to touch with an “electric” type of pain. She had previously come to the same ED with the complaint of abdominal pain at which time she did not receive a definitive diagnosis, and the rash erupted two days later. Thus far, she had minimal relief with over-the-counter pain medication. She denied ever having a similar rash in the past or any known sick contacts. She did endorse having the “chicken pox” as a child. She had been in her previous state of health prior to these symptoms arising, but did note she recently had surgery on her left upper extremity. She denied any additional symptoms such as fever, chills, cough, shortness of breath, vision changes, nausea, vomiting, or diarrhea.

**Figure 1:**
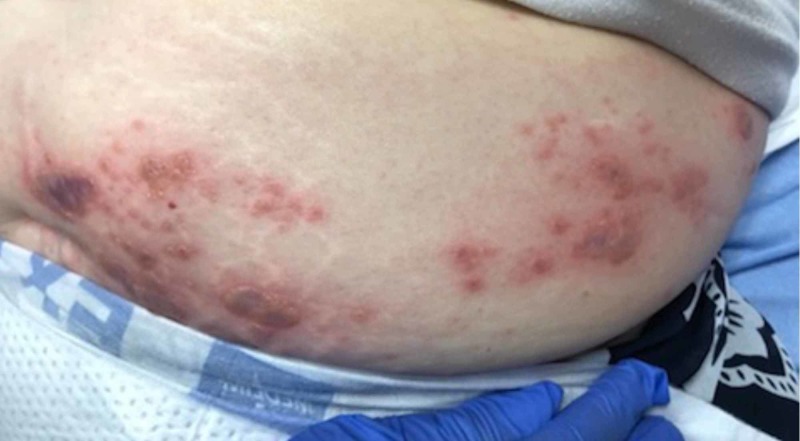
Lesions on left lower abdomen

**Figure 2:**
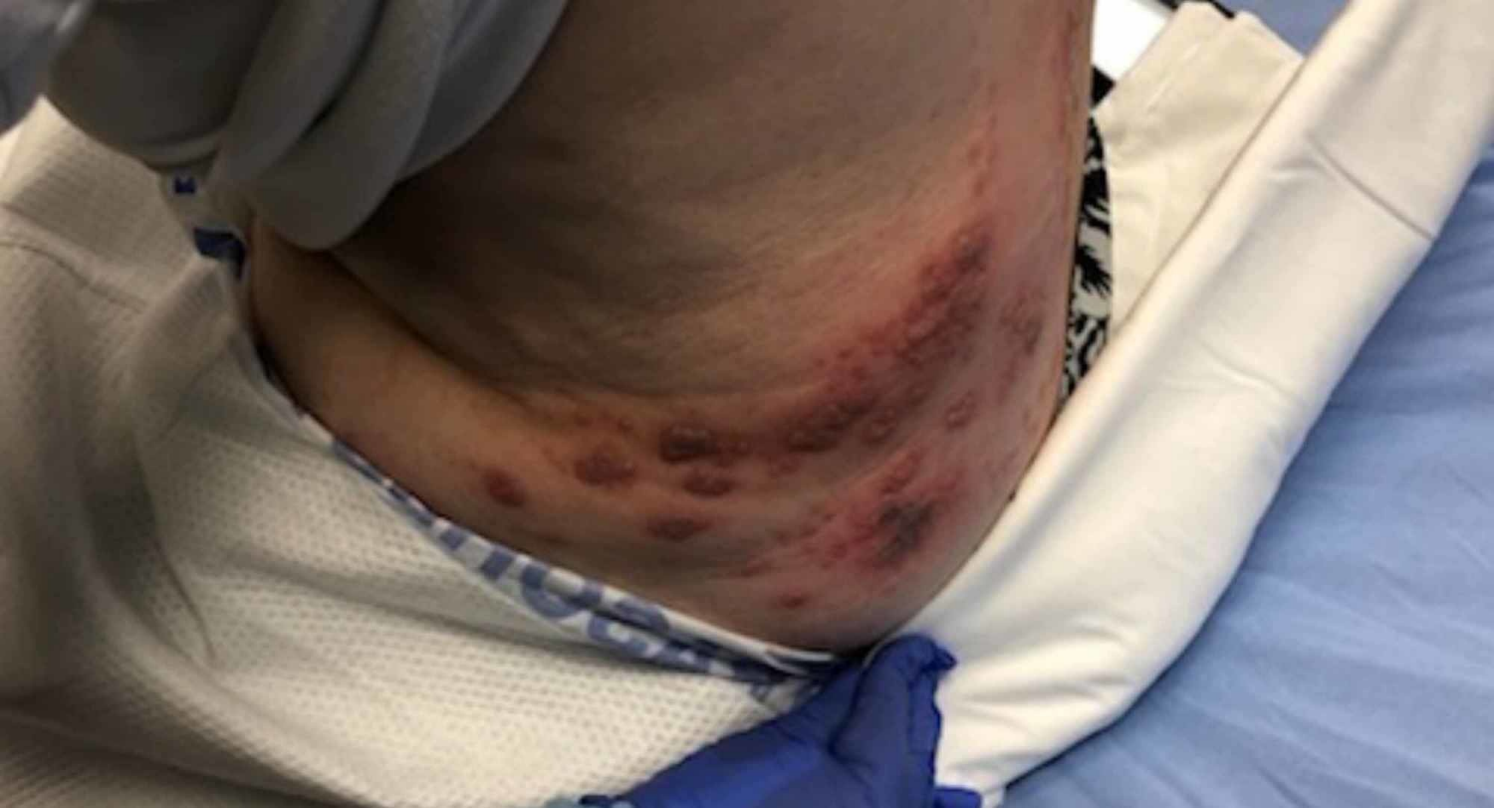
Lesions wrapping around to left flank and back

**Figure 3:**
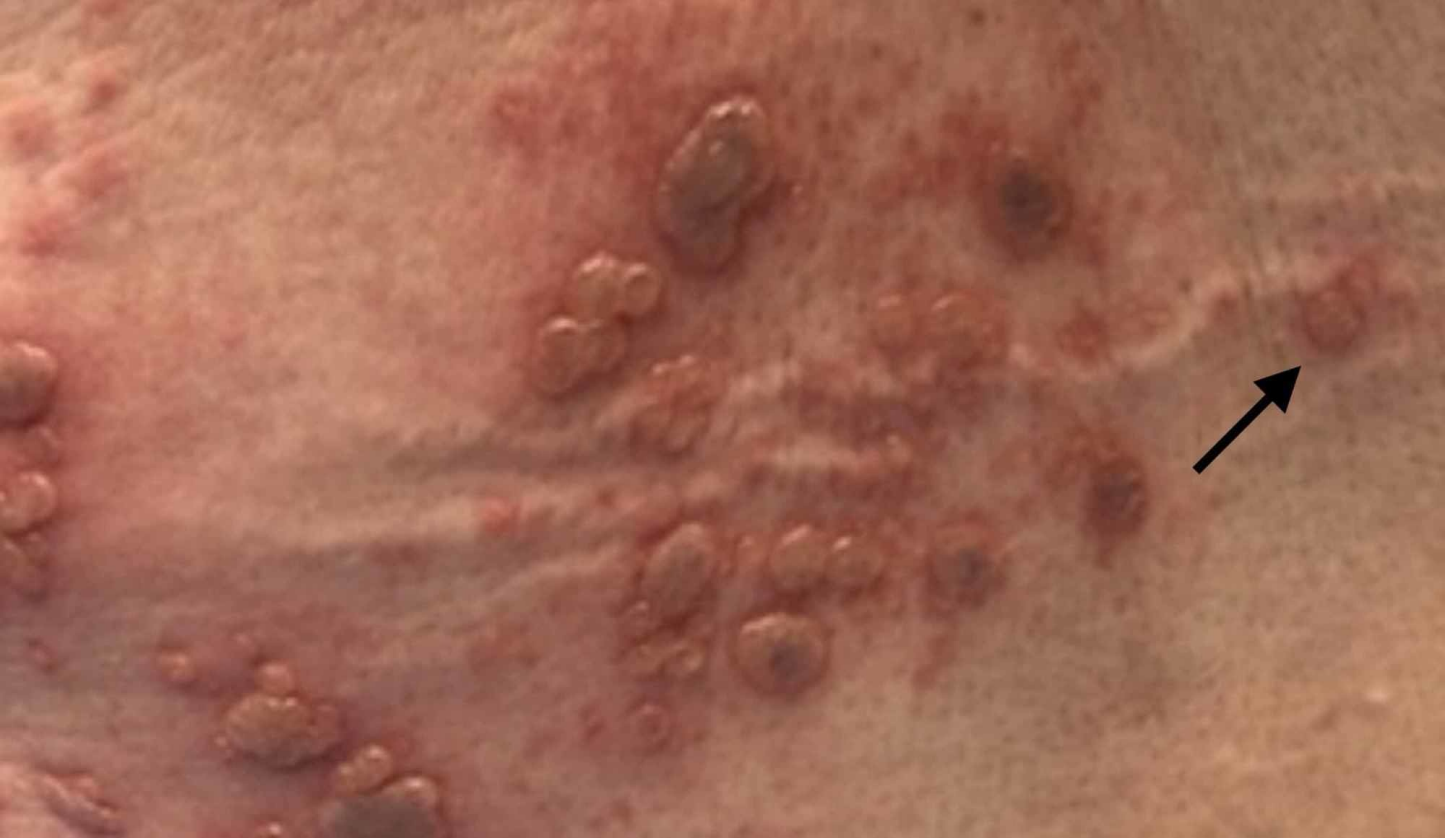
Lesions on left flank and back crossing midline

**Figure 4:**
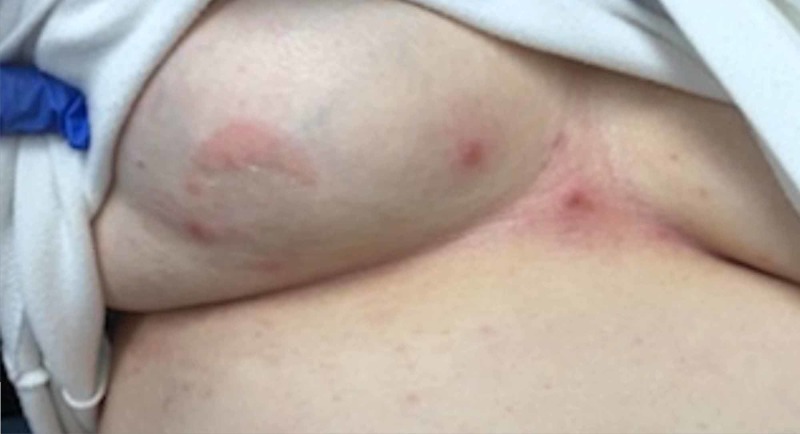
Lesions on anterior chest and left breast

**Figure 5:**
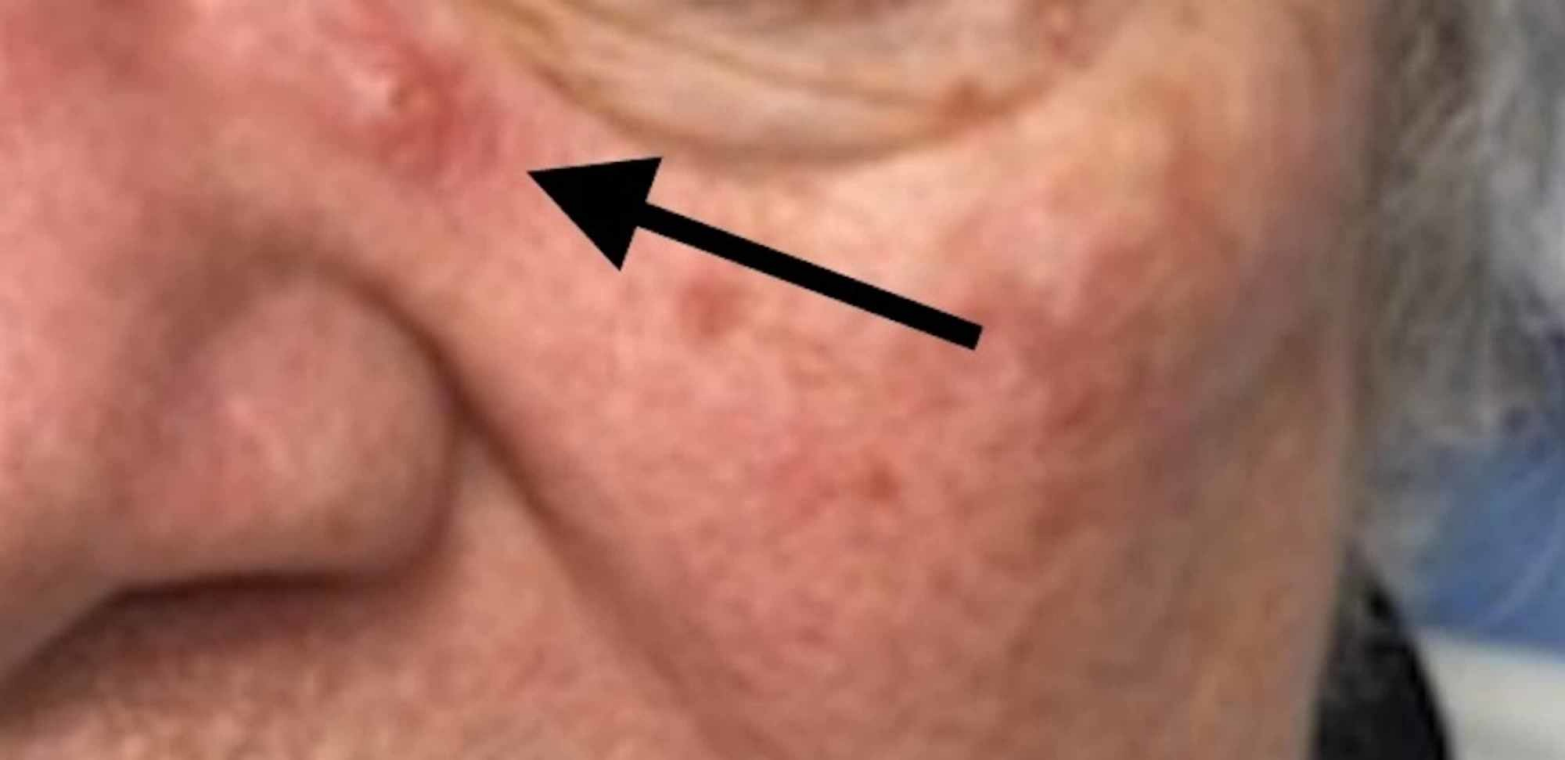
Lesion under left eye

On physical examination, the patient’s vital signs were normal other than mild hypertension. Abdominal examination included a vesicular, erythematous rash with all lesions in the same stage of development located over the left lower abdomen (Figure 1) wrapping around to the left flank (Figure 2), with a few lesions crossing the midline (Figure 3). She also had the same lesions on the right anterior chest and right breast (Figure 4) with scattered lesions into the right anterior axillary line. A single lesion was located below the left eye (Figure 5), and another single lesion on the right side of her chin. The rash was extremely tender to light palpation with associated paresthesias. Fluorescein dye was applied to both eyes to examine for potential signs of herpes zoster ophthalmicus, and no such lesions were noted. The nose and both ears including the acoustic meatus were lesion free. Neurological examination was normal. On completion of the physical examination, at least four discrete dermatomes were found to be involved. The patient was placed under contact and airborne precautions.

**Figure 6:**
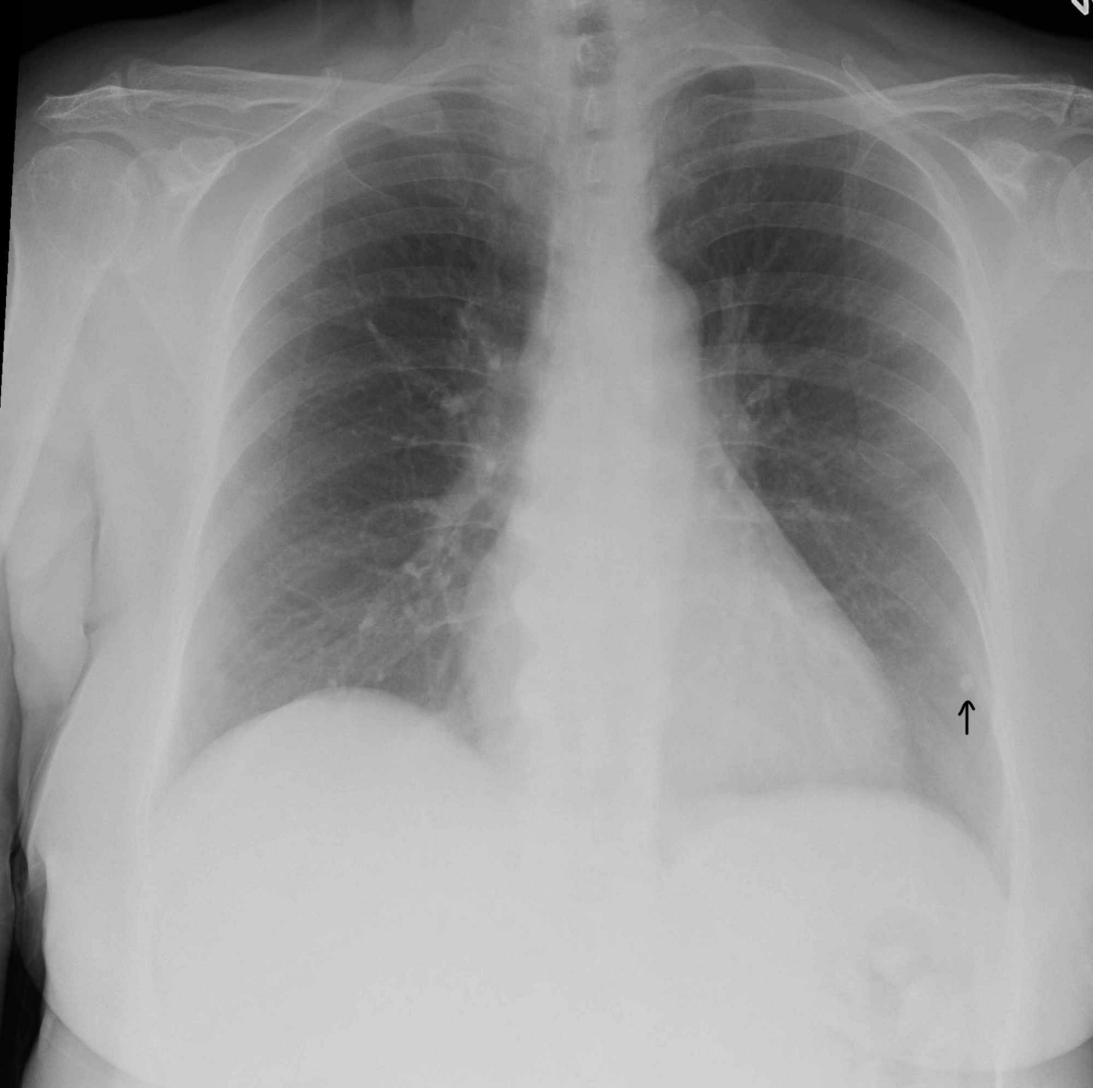
Chest radiograph demonstrating stable calcified granuloma (arrow)

The patient’s complete blood count was negative for a leukocytosis or leukopenia, but was notable for the presence of atypical lymphocytes. Chemistry panel was unremarkable except for hyperglycemia. Chest radiography revealed a 3 mm calcified granuloma along the periphery of the left lower lobe (Figure 6), which the patient was previously aware of. No acute findings were otherwise noted.

At this time, the patient was started on intravenous (IV) acyclovir with concern for disseminated herpes zoster and given morphine for acute pain control. Our findings and concerns were discussed with the patient who agreed with a plan for admission for further treatment, evaluation for possible immunocompromised state, and pain control. On admission, the plan for infectious disease (ID) consultation was discussed with the medicine team. Due to the unusual presentation of disseminated zoster in an immunocompetent patient, IV acyclovir was continued by ID with ongoing investigation into possible human immunodeficiency virus infection, underlying malignancy, hepatitis infection, and other autoimmune conditions. Through day 3 of her hospitalization, she continues to develop additional lesions, notably on her right upper quadrant and left ear. She also developed an acute kidney injury during her hospitalization which was likely crystal induced and resolved after IV acyclovir administration was changed from every eight hours to every 24 hours. The patient’s lesions began to crust over and resolve on day 4 of hospitalization and on day 5 she was transitioned to oral acyclovir and discharged from the hospital.

## Discussion

Herpes zoster is an opportunistic reactivation of herpes varicella that occurs when the immunity of previous inoculation or native immune response wanes. Advancing age is the one of the most important risk factors for this reactivation, particularly in the immunocompetent patient [3,4]. However, as seen in this case, a thorough physical examination may uncover one of the many possible complications of the disease. This patient eventually had at least five dermatomes involved on both sides of the body by the end of her hospitalization with several satellite lesions with some crossing the midline, indicating true disseminated disease and likely hematogenous spread of the virus. This highlights the importance of considering the possibility of disseminated disease and other complications when approaching the fairly simple diagnosis of zoster of an immunocompetent patient in the ED. We can highlight the importance of a thorough ophthalmic examination for possible herpes zoster ophthalmicus or acute retinal necrosis with the possibility of permanent vision loss and severe decline in quality in life, as well as chest radiography to evaluate for associated pneumonitis that would greatly increase the mortality of the disease. Otoscopic and neurological examination are other important aspects to be sure to include in the examination to evaluate for cranial nerve involvement in the case of possible Ramsay Hunt syndrome [5]. These are all fairly quick physical examination aspects, but serve to provide reassurance and a safe disposition for a patient to be treated with oral medication at home in cases of uncomplicated zoster in the immunocompetent patient.

For this patient, however, even with a reassuring physical examination other than significant and disseminated cutaneous involvement, it was important to further evaluate her in the inpatient setting for potential causes of underlying immune compromise. While undergoing initial IV therapy with acyclovir, she was tested for various immune compromising conditions and was found to be positive for rheumatoid factor. She was negative for HIV, hepatitis, and antinuclear antibody screening tests. Additionally, computed tomography (CT) imaging of the chest was performed to further evaluate for underlying lung malignancy, given the known lesion that was noted on chest X-ray with no other lesions found on CT. A proposed possible cause of reactivation and disseminated disease in this patient was her recent left upper extremity surgery as well as rheumatoid arthritis (although the patient had never been symptomatic in the past). With closer, inpatient evaluation and treatment, the patient was able to be observed for further complications such as bacterial superinfection of her lesions which she could have been more susceptible to if a more serious cause of immune suppression had been uncovered. This patient had already been discharged from the ED once after the onset of symptoms prior to the eruption of her rash, and with a 2% incidence of disseminated zoster in the immunocompetent, this was an appropriate case to assume the worst and hope for the best of outcomes.

## Conclusions

This case serves as a reminder for the careful physical examination in patients with the usually simple, clinical diagnosis of herpes zoster. The most common complication will continue to be post-herpetic neuralgia. However, the more serious complications of the disease should be carefully considered by history taking and physical examination in each patient presenting with herpes zoster regardless of their immune status. These severe complications will continue to be more common in the immune compromised population but cannot be ignored in the general population.
